# Compact Paper-Based Quasi-Solid-State Organic Electrochemical Transistor (QSS-OECT) for Sensing Hydrogen Peroxide

**DOI:** 10.1021/acsaelm.5c00559

**Published:** 2025-07-31

**Authors:** Andrés Alberto Andreo Acosta, Pascal Blondeau, Francisco Javier Andrade

**Affiliations:** Department of Analytical Chemistry, 16777 Universitat Rovira i Virgili, Carrer Marcel·lí Domingo, 1, 43007 Tarragona, Spain

**Keywords:** Organic electronics, OECT, paper-based biosensors, solid-state electrochemical cells, PEDOT:PSS, hydrogen peroxide

## Abstract

Organic electronics have garnered important attention, as they offer alternatives to classic, more expensive electronic materials and great biocompatibility for building (bio)­chemical sensors. Among them, organic electrochemical transistors (OECTs) provide *in situ* signal amplification, which translates to excellent performance in electrochemical (bio)­sensing. This work presents a compact paper-based thick-film quasi-solid-state OECT (QSS-OECT) that integrates the gate and channel in a vertically stacked design. Electrochemical activation by pulsing the gate voltage allows reaching high transconductances of up to 12.4 mS. This system is applied to the detection of H_2_O_2_ with a sensitivity of 3.5 ± 0.3 mA/dec. The compact vertical configuration of the device allows for a drastic sample volume reduction, which can be performed with droplets of 1 μL as well as under the flow regime with an open cell configuration. The versatility and high transconductance of this device open new avenues for integration within low-cost sensors and biowearable platforms.

## Graphical abstract

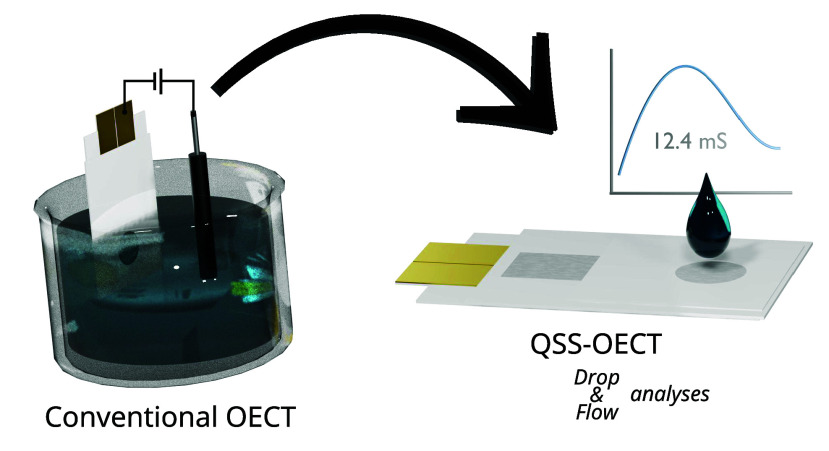

## Introduction

Organic electrochemical transistors (OECTs) are gaining relevance in chemical analysis as they are versatile platforms that can be used to create highly sensitive (bio)­chemical sensors.^1^ OECTs can be built on flexible substrates, such as paper,^2^ plastics,^3^ and textile fabrics,^4^ and can be operated under flow conditions.^5,6^ This versatility also extends to different configurations, such as horizontally and vertically stacked designs,^7,8^ and different manufacturing approaches, such as inkjet and screen-printing,^9^ electrodeposition,^10^ and even 3D-printing.^11^ OECTs show exceedingly high transconductance values at low operating gate voltages—typically below 1 V. Thus, they show key advantages such as low power consumption, reduced risk of interferences, and minimal issues related to side reactions, such as water splitting and corrosion.

Like other transistors, OECTs work with a three-electrode setup, namely, the source (S), drain (D), and gate (G). S and D electrodes are connected through the semiconducting channel (Ch), which is made of an organic conducting polymer, typically poly­(3,4-ethylenedioxythiophene)-poly­(styrenesulfonate) (PEDOT:PSS). The G electrode and the channel are connected through an electrolyte solution. This is a major difference in the case of the OECTs with respect to other devices, such as the field-effect transistors, which use a dielectric material as a barrier to isolate the semiconducting channel from the solution. In OECTs, the direct contact with an electrolyte solution allows the free exchange of ions, something that is intrinsically related to its working mechanism.

The principle of operation of OECTs has been extensively studied.^12,13^ In short, a fixed voltage is applied between S and D (the drain voltage, *V*_*D*_) to generate an electronic current that flows through the channel (*I*_*D*_). The value of *I*_*D*_ depends on the electrical conductivity of the channel, which is determined by the doping state, i.e., the fraction of the conductive polymer that is in its highly conductive, oxidized form (PEDOT^+^) with respect to the reduced, nonconductive form (PEDOT^0^). The fundamental principle of OECTs is that this doping state is controlled by the presence of cations. The conductive form (PEDOT^+^) is stabilized by the electrostatic effect produced by the negatively charged sulfonate groups (PSS^–^). Cations incorporated into the channel disrupt this stabilizing effect, promoting the transition to the nonconductive form (PEDOT^0^),^14^ i.e., dedoping the conductive polymer:

1 $${PEDOT}^{+}\text{:}{PSS}^{-}+M_{\left(aq\right)}^{+}+e^{-}↔{PEDOT}^{0}+M^{+}\text{:}{PSS}^{-}$$

For a constant *V*_*D*_, changing the channel electrical conductivity results in a change in *I*_*D*_. To control the migration of cations from the solution into the channel, a voltage is applied between the G and S electrodes (the gate voltage, *V*_*G*_). As a result, *I*_*D*_ becomes a function of *V*_*G*_. This is the essence of the voltage-to-current transduction mechanism of the OECTs, which is measured by the transconductance (*g*_*m*_),

2 $$g_{m}=\frac{\partial I_{D}}{\partial V_{G}}$$

Due to the high difference in conductivity between the two forms of PEDOT, small changes in the gate voltage produce large changes in channel current. Furthermore, one of the most unique characteristics of the OECT is that their change in channel conductivity is not a surface phenomenon but occurs in the whole volume of the channel. This is usually described in Bernards model as the volumetric capacitance C*,^13^ and it is the reason why OECTs display *g*_*m*_ values that are significantly higher than for other type of transistors. For (bio)­chemical sensors, this high-power amplification of the signal results in systems with high sensitivity. In a previous work, we have exploited this characteristic through the generation of thick-film OECT biosensor.^15^

Since *I*_*D*_ follows changes in *V*_*G*_, biosensors are often built by functionalization of the gate. Therefore, (bio)­chemical events altering the *V*_*G*_ are turned into a change in *I*_*D*_. Using different materials and nanomaterials, this approach has been applied to the detection of a plethora of biomolecules.^14^ Alternatively, detection based on the electrocatalytic properties of the gate has been exploited. For example, the open circuit potential of Pt electrodes coated with a polyelectrolyte, such as Nafion, responds to the presence of hydrogen peroxide.^16^ Therefore, OECTs with a Pt-Nafion gate are very effective sensors of this molecule. Thus, OECTs with Pt-Nafion gates can be used for the detection of peroxide, and with a suitable oxidase enzyme, they can be turned into biosensors for molecules such as glucose, lactate, and ethanol.

A downside of the OECT sensors based on changes of *V*_*G*_ is that ohmic and capacitive losses in the vicinity of the gate may lead to a weak effective *V*_*G*_, which results in a poor transconductance. In these cases, the functionalization of the channel interface instead of the gate can be used.^14^ This approach has been successfully applied to the detection of biomolecules and, through the addition of an ion-selective membrane, has been also used to create ion-sensing OECTs.^17,18^ These systems show an enhanced performance, and since a generic gate can be used, they can create compact multiplexed sensors.

Simplification of the OECT design and reduction of its overall footprint to fit into paper-based test strips, wearable, and embedded systems is increasingly required to develop devices for point-of-need applications. In these scenarios, the high dependence of the gating mechanism on the electrolyte solution may hinder the progress. Replacing liquids with solid- or quasi-solid-state electrolytes to create more compact and robust sensors is an attractive avenue. For example, we have recently reported a novel design of electrochemical sensors in a vertically stacked configuration using paper-based Pt electrodes separated by a layer of an ionomeric material.^19^ This device can detect hydrogen peroxide with outstanding performance even when using sample volumes below 1 μL.

Based on a similar concept, this work explores the development of a quasi-solid-state ionomeric material to build OECTs for biosensing applications. A thick-film PEDOT:PSS channel is used to maximize the sensitivity. Additionally, the gate is integrated on top of the channel using a layer of Nafion as a separator. This polyelectrolyte shows high proton conductivity and mechanical and chemical stability.^20^ For this reason, it is widely used as a solid polymer membrane separator in fuel cells, batteries, capacitors, and other electrochemical devices.^21–24^ Interestingly, the use of Nafion in OECTs is scarcely reported, mostly used as a membrane separator in systems using liquid solutions^25,26^ or as solid polymer electrolyte (SPE) in neuromorphic devices.^27^ Han et al. have reported the use of a polyelectrolyte material in contact with the PEDOT:PSS to build ion-selective electrodes.^28^ In this design, the polyelectrolyte acts as an internal ion reservoir that is isolated from the solution by the polymeric membrane and provides the ions for the OECT gating mechanism. Using a similar principle, this paper explores the use of a layer of Nafion in contact with the PEDOT:PSS channel to create a novel paper-based quasi-solid-state OECT (QSS-OECT). In this case, the Nafion is the ion reservoir that provides ions to the channel and is electrically connected with the solution through the pores of the paper. This device is fabricated with a facile handcrafted approach in a vertically stacked configuration and is used for the detection of hydrogen peroxide. The operation of this device in single droplet assays as well as under a flow regime with outstanding performance and using low sample volumes is demonstrated. The system shows a promising performance for the detection of hydrogen peroxide, which is the gateway for the fabrication of a myriad of direct and labeled bioassays. Demonstration of the use of this device in drop and flow systems shows the potential to become a platform for future biosensing applications.

## Experimental Section

### Reagents and Materials

All reagents employed are analytical grade and were purchased from Sigma-Aldrich (Merck, Spain). They were used without further purification, with the exception of the 3–4% aqueous solution of high-conductivity-grade PEDOT:PSS, which was filtered once by means of poly­(ether sulfone) 0.45 μm Millex syringe-driven filters (Merck, Spain). A 5% solution of Nafion 117 perfluorinated resin in a mixture of lower aliphatic alcohols and water was cast as the solid-state electrolyte on the back of the gate electrode, as well as over its window. A 30% (*v*/*v*) H_2_O_2_ stock solution was used to prepare daily fresh standard solutions of peroxide. Also, a 0.1 M phosphate-buffered saline solution (PBS, pH 7.4) was prepared by dissolving suitable amounts of Na_2_HPO_4_, KH_2_PO_4_, NaCl, and KCl, adjusting to the final pH. All solutions were made using Mili-Q deionized water (MilliporeSigma, Burlington, MA, USA).

### Instruments and Software

TENMA (Element14, Newark, NJ, USA) or KEYSIGHT E3631A (Keysight Technologies, Santa Rosa, CA, USA) DC laboratory power supplies were used to power the transistors. The channel current (*I*_*D*_) was measured using a KEITHLEY 2100 multimeter (Tektronix Instruments, Inc., Malvern, PA, USA). Galvanostatic charge/discharge (GCD) tests were performed with an Autolab PGSTAT128N potentiostat/galvanostat (Metrohm AG, Herisau, Switzerland). An InfusionONE Programmable Syringe-Pump system (New Era Pump Systems, Inc., Farmingdale, NY, USA) was used for the flow experiments.

Paper-based electrodes were built by sputtering metals onto the substrate. Sputtering was carried out with an AJA ATC Orion RF magnetron (AJA International, MA, USA). Instrumental parameters were adjusted to produce a 100 nm layer of the metallic material on the paper.

### QSS-OECT Building and Activation

#### Transistor Fabrication

The first step in the fabrication of the transistor is the making of paper-based channels. Details on this procedure have been reported in a previous work.^15^ In short, a 0.5 mm-wide adhesive strip (ABC Hobby Co., Osaka, Japan) was placed over a matte photography-quality paper (weight of 200 g/m^2^), and then, a 100 nm Au layer was sputtered over this assembly. Removing the adhesive tape creates two Au tracks—the S and D electrodes—separated by a 0.5 mm gap, which will be the channel length (layers S and D in Figure 1b). This system was cut into 23 mm-long pieces and sandwiched between two hydrophobic adhesive masks (UPM Raflatac, Helsinki, Finland). A 1.5 mm-radius circular orifice in the top mask leaves partially exposed the Au pads separated by a 0.5 mm gap. The channel is made by drop casting 3 μL of the filtered PEDOT:PSS solution to completely cover this window (layer C in Figure 1b). This system is first dried at room temperature and then in an oven (100 °C) for 20 min. Finally, to waterproof the system, the perimeter of the window was sealed with cyanoacrylate glue.

**Figure 1:**
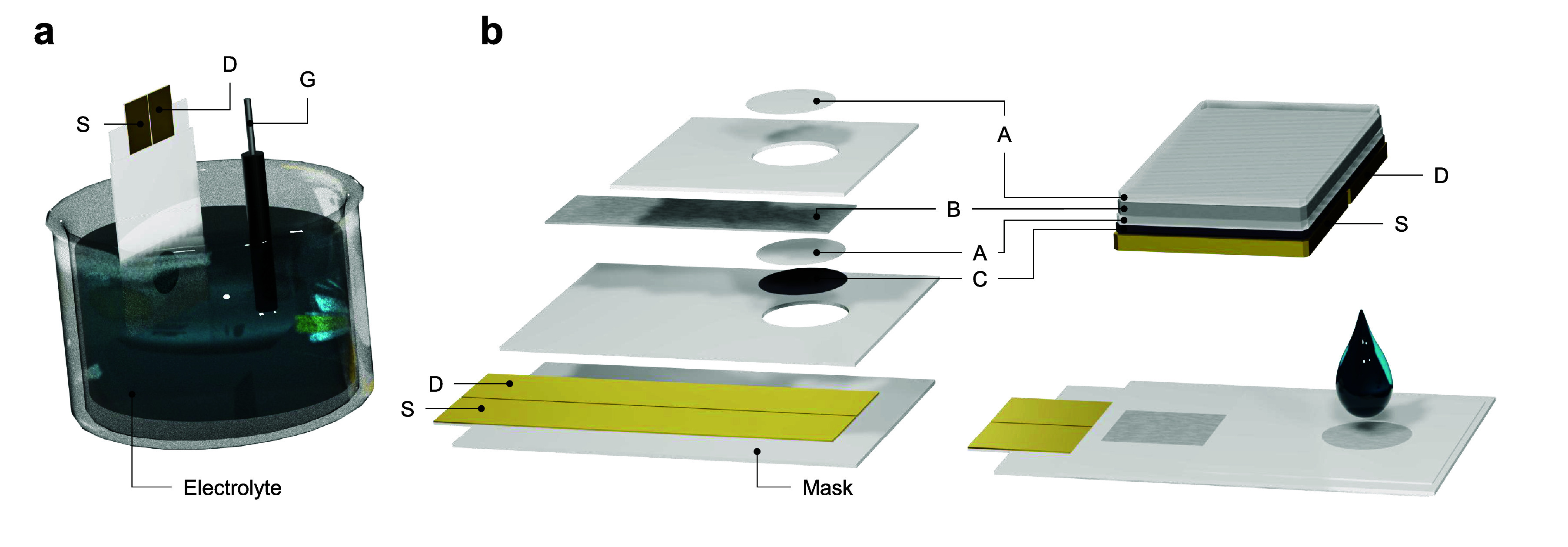
Schematics of the LS-OECT (a) and QSS-OECT (b) approaches. A: Nafion, B: Pt gate, C: conductive polymer (PEDOT:PSS), D: Au drain, S: Au source, and G: Ag/AgCl gate.

As a second step, gate electrodes were built and integrated into the channel. Gate electrodes were made by sputtering a 100 nm layer of Pt on a filter paper (Whatman Grade 1, GE Healthcare, Chicago, IL, USA), which was then cut into 6 × 16 mm strips (layer B in Figure 1b). Then, 7 μL of Nafion was drop cast on the backside (nonmetallized side) of these strips (bottom-A layer in Figure 1b), which were then placed on top of the channel (with the metallized Pt side facing outward). Once dried, an adhesive mask with a circular orifice (1.5 mm radius) was placed on top of this electrode to fix the gate and create an electrochemical window. As a final step, 3 μL of Nafion was drop cast over the Pt of the exposed window (top-A layer in Figure 1b). This ensemble was dried overnight at room temperature. For comparison purposes, control devices were fabricated in a similar way but without the addition of Nafion on the back of the gate. Also, conventional OECTs with the gate and channel separated by a liquid solution (LS-OECT) were tested. Schematics of these devices can be seen in Figure 1. Assembly dimensions are provided in the top view displayed in Figure S1.

#### QSS-OECT Activation

In the development of electrochemical sensors, it is common that devices are not used “as built”, but that they require a preconditioning as a last step of the fabrication process. To evaluate this point, different chemical and electrochemical strategies were tested: 1) using nonconditioned (NC), as-built QSS-OECT, 2) overnight conditioning with sensors immersed in PBS while powered at the operational voltages (−0.4 V_*D*_, 0.5 V_*G*_), 3) chemical conditioning (CC*n*, with *n* ^th^ sensing test); the QSS-OECT was kept overnight immersed in PBS (unpowered), and prior to use the sensor was immersed for 20 min in 10 mM H_2_O_2_ under operational voltages (−0.4 V_*D*_, 0.5 V_*G*_), 4) pulse activation (PA) by pulsing *V*_*G*_ with a square wave (0 to 3.3 V) at 1 Hz and 80% duty cycle during 20 min, with the sensor immersed in PBS. The pulse parameters were optimized to the minimum power required to reach the off-state in QSS-OECTs during pulse activation.

#### Electrochemical Measurements and Setup

Current measurements were performed with the transistor immersed in a 5 mL cell with PBS, unless stated otherwise. Transconductance and transfer curves were obtained by sweeping *V*_*D*_ (from −0.04 to −0.40 V, in steps of 0.06 V) and *V*_*G*_ (0.1 to 0.8 V, in steps of 0.1 V), respectively.

Characterization using galvanostatic charge/discharge (GCD) experiments was performed between channel and G with the Autolab PGSTAT128N potentiostat/galvanostat. The voltage window was set from −0.1 up to 0.6 V with a galvanostatic current of ±1 μA applied upon 10 charge/discharge cycles.

For the flow-based measurements, the PBS carrier solution was dispensed using the InfusionONE syringe-pump at a flow rate of 50 μL/min. Standard solutions in PBS were injected with a six-port injection valve. For measurements with individual droplets, 1 μL of standard solutions in PBS was added directly onto the gate window. Three consecutive washings with 15 μL of PBS and drying with tissue paper were performed between the additions.

## Results and Discussion

### Electrical and Electrochemical Characterizations of the QSS-OECT

Figure 1 compares the design of conventional LS-OECTs and the QSS-OECTs. In the conventional system, the G electrode is separated from the channel by a liquid electrolyte. In QSS-OECTs, on the other hand, the gate and the channel are separated by a layer of hydrated Nafion. This polyelectrolyte does not affect the dynamic resistance of the QSS-OECT channel, which shows ohmic behavior with a resistance of 20.8 Ω (Figure S2).

A first set of experiments was conducted to compare the response of the conventional and QSS-OECT. Transistors were immersed in PBS, and their transfer curves (*I*_*D*_ *vs V*_*G*_, at constant *V*_*D*_) were evaluated to calculate the transconductance (*g*_*m*_) of each type of OECTs (Figure S5). The response of the conventional (nonconditioned) LS-OECT (Figure 2A) is similar to what has been already reported.^13^ As *V*_*D*_ increases, the maximum *g*_*m*_ is found at lower *V*_*G*_ values. A maximum *g*_*m*_ of 9.8 mS is obtained at *V*_*G*_ = 0.4 V (*V*_*D*_ = – 0.4 V). For a nonconditioned QSS-OECT, a significantly different pattern is obtained (Figure 2B). In this case, *g*_*m*_ grows monotonically reaching a maximum value (*g*_*m*_ = 9.2 mS) at the highest *V*_*G*_ tested (0.8 V). These differences suggest that the sensor geometry and the use of a polyelectrolyte have an influence on the gating efficiency. It should be remembered that the OECTs operate under two gradients of electrical potential: from one side *V*_*D*_, which drives electrons along the channel, and from the other *V*_*G*_—orthogonal to *V*_*D*_—which forces the migration of cations into and out of the channel. Some experiments were performed to evaluate whether, due to the proximity between the channel and gate in the QSS-OECT, there could be some interference between *V*_*G*_ and *V*_*D*_. The results show that there is no significant effect (Figure S6).

**Figure 2:**
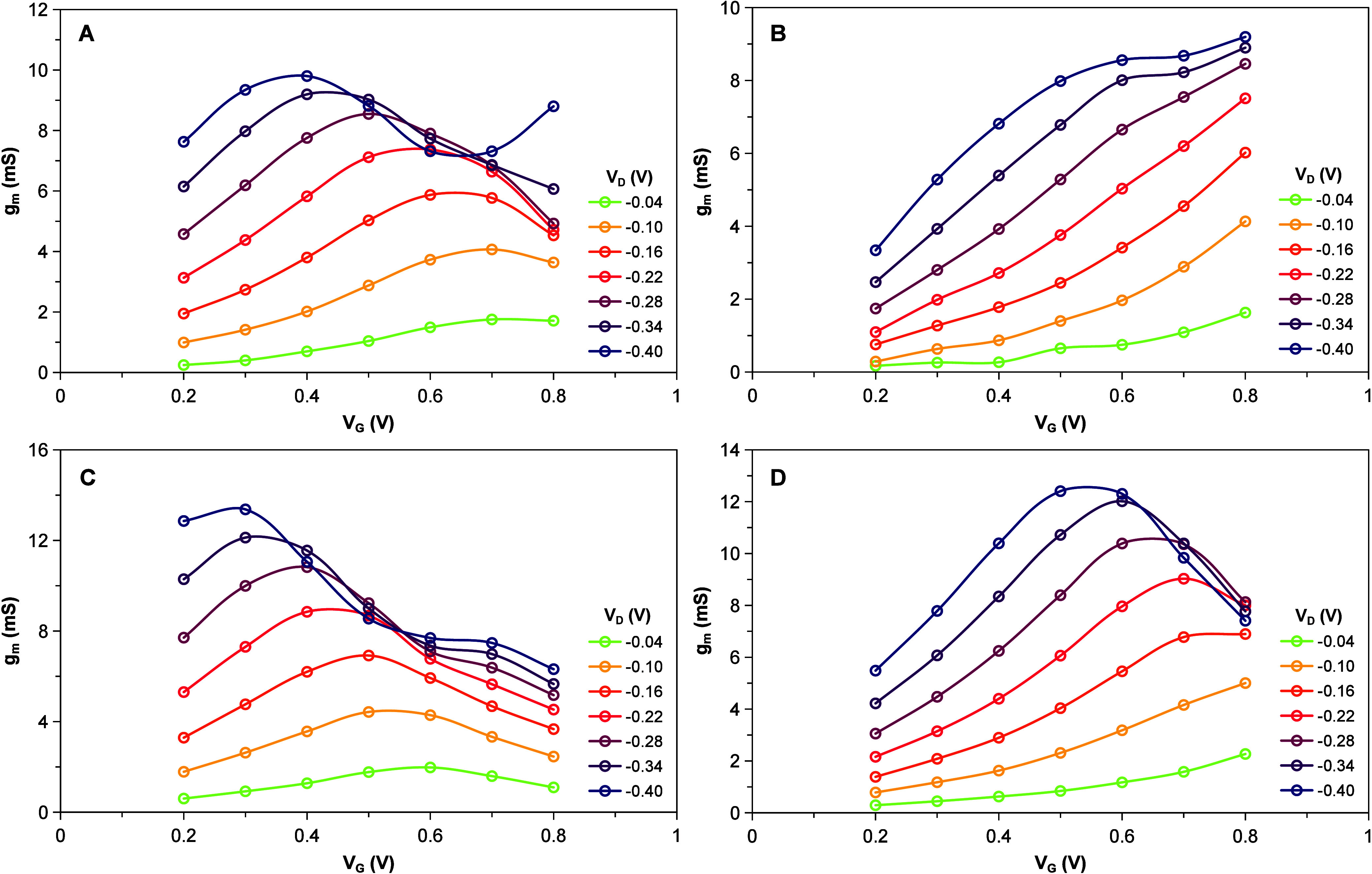
Transconductance profiles for different OECT configurations in PBS: LS-OECT (A), unconditioned QSS-OECT (B), chemically conditioned QSS-OECT by means of H_2_O_2_—first use, CC1—(C), and pulse-activated QSS-OECT (D). A separated Pt/Nafion gate electrode was used for the characterization of the LS-OECT (A).

The data in Figure 2B show that the results for the QSS-OECT are not optimum, since in this type of chemical sensors, *g*_*m*_ should be maximized at the lowest possible *V*_*G*_, so that the risk of interferences is reduced. The fact that the maximum *g*_*m*_ in the solid-state devices is reached at higher *V*_*G*_ suggests a less efficient transfer of energy to the ions. This phenomenon can be caused by factors affecting the gate electrochemical interface, system geometry, electrolyte composition, etc., since capacitive and resistive losses can reduce the intensity of the local electrical field on the vicinity of the ions. Regarding the gate, the Pt–Nafion system has been studied for many decades, as the reactivity of this triple metal–water–polymer interface is the result of many interdependent factors, such as the Pt surface composition, the polymer structure on the metal surface, and the water activity in the channels.^29,30^ It is well known that the generation of a surface oxide layer on Pt increases the reactivity toward peroxide and changes the electrical characteristics of the interface.^31^ This oxide layer can be created by chemical as well as electrochemical methods. It has been shown that peroxide reacts on Pt, generating surface-adsorbed oxygenated species. Alternatively, the activation of the Pt surface using controlled potential methods has been demonstrated.^32,33^ Therefore, to reduce the optimum *V*_*G*_ of the QSS-OECT, chemical and electrochemical surface conditioning treatments were explored. It is well known that the electrocatalytic activity of Pt toward peroxide depends on several factors, such as the surface oxidation state (PtO), adsorbed species, etc.^30^ Thus, different conditioning methods can affect the surface reactivity of the gate, influencing the response of the QSS-OECT. First, conditioning with a 10 mM peroxide solution for 20 min under typical operating voltages (*V*_*G*_ = 0.5 V, *V*_*D*_ = – 0.4 V) was evaluated. After this treatment, the sensor is washed, and the transconductance is evaluated, observing a significant change on the profiles (Figure 2C), which become more similar to the LS-OECT (Figure 2A). The maximum *g*_*m*_ (13.4 mS) is reached at *V*_*G*_ = 0.3 V (for *V*_*D*_ = – 0.4 V). It has been shown that peroxide reacts on Pt surfaces, either through the spontaneous oxidation on PtO sites or through the adsorption on pristine Pt sites.^34^ Thus, it can be assumed that changes produced by these reactions on the electrode surface result in more effective gating. This chemical conditioning confirms that modification of the electrode surface is a way to optimize the transistor performance. However, while effective, it is not desirable from a practical standpoint. Instead, it has been shown that the surface reactivity of Pt can be also electrochemically modified.^35^ Preliminary work using different time-potential schemes (data not shown) has shown that relatively high voltages at a frequency of 1 Hz produce the best results. To prove this point, an activation through this time-potential scheme was tested. The result of these experiments (Figure 2D) shows that after this treatment the gating behavior changes and becomes more similar to the response obtained with the conventional transistors (Figure 2A). After this treatment, *g*_*m*_ reaches a maximum of 12.4 mS at a *V*_*G*_ = 0.5 V (for *V*_*D*_ = – 0.4 V).

### Detection of H_2_O_2_

After optimization of the transconductance of the transistor in PBS, preliminary experiments were performed to evaluate the response to H_2_O_2_. These results (Figure S7) show that the sensitivity toward peroxide follows a similar pattern to the transconductance in PBS, with a response that increases as *V*_*G*_ increases. Similar results have been previously reported for conventional OECTs and are due to the influence of the electrochemical reactions of oxygenated species on the effective gate voltage.^15^ Normalized responses (see inset of Figure S7) show an optimal value of transconductance at *V*_*G*_ close to 0.6 V (for *V*_*D*_ = – 0.4 V), which is consistent with the results of the transconductance in PBS. Thus, the detection of hydrogen peroxide using the QSS-OECTs under different conditions was evaluated at *V*_*G*_ = 0.5 V and *V*_*D*_ = – 0.4 V.

Experiments were then conducted to evaluate the effect of a conditioning treatment on the response to peroxide. The time traces and corresponding calibration curves for the detection of peroxide after different conditioning are shown in Figure 3A and B, respectively. First, an as-built, nonconditioned device was tested. In this case, a well-defined profile is obtained only at low concentrations, i.e., with an initial addition of 30 μM. While this confirms that the device responds to peroxide, as the concentration increases, the profiles become more erratic, without clear variations after the additions. Attempts to stabilize the transistor in PBS for longer times do not improve this result. Interestingly, the time trace of Figure 3A is for a device that is calibrated for the first time (CC1 series). After successive calibrations, the response becomes increasingly stable and well defined (Figure S8), suggesting that the addition of peroxide produces some modification on the OECT that improves the response. For a third calibration (CC3), for example, the time trace shows very clear changes upon additions of peroxide over the whole range. This suggests that several treatments with an increasing concentration of H_2_O_2_ followed by washing cycles improve the sensor performance. After this third treatment, no significant improvement is observed (Figure S8 and Table S1).

**Figure 3:**
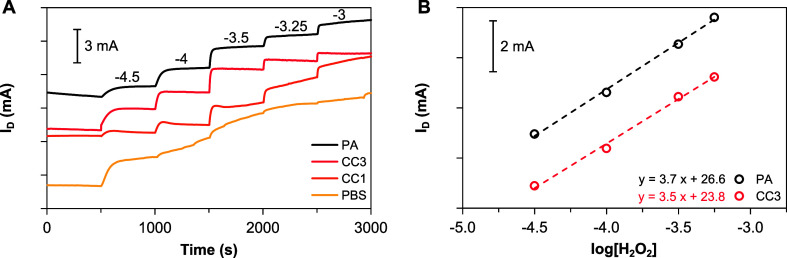
*I*_*D*_ time traces (A) and calibration curves (B) for the detection of H_2_O_2_ at *V*_*G*_ = 0.5 V and *V*_*D*_ = – 0.4 V using QSS-OECTs with different conditioning approaches (offset): PA (pulse-activated), CC3 (chemically conditioned by means of H_2_O_2_, third consecutive run), CC1 (chemically conditioned by means of H_2_O_2_, first test), PBS (conditioned with PBS).

Since this enhancement obtained through chemical conditioning is impractical in real scenarios, attempts to emulate the chemical conditioning through an electrochemical process were explored. It is well known that prolonged application of constant voltages on Pt electrodes leads to the generation of subsurface oxide layers, which can irreversibly affect the electrode response. For this reason, conditioning through electrical pulses was evaluated. Pulsing conditions are widely used in the operation and testing of industrially manufactured electronics.^36–38^ In the case of OECTs, voltage pulses are mainly utilized to perform stability assessments,^39,40^ characterizations,^41^ synapse modeling and control,^42,43^ or even enhance the interactions in the sensing of antigens for disease detection and cells monitoring.^44,45^ In this work, pulsing the gate is aimed at activating the sensor. Thus, the effectiveness of different time-voltage schemes was evaluated. The results show that the pulse activation has a positive effect on the signal (Figure 3), with optimum results obtained with a square wave of amplitude from 0 to 3.3 V at 1 Hz and 80% duty cycle for 20 min in PBS (*V*_*D*_ was kept at −0.4 V). Noteworthy, while three runs were necessary with the chemical activation, only one step of this pulse activation is enough to enhance the performance. The use of this pulse activation modifies the transconductance profiles (Figure 2D), reaching a maximum value of 12.4 mS at *V*_*G*_ = 0.5 V. Figure S9 shows these transconductance profiles of the fully activated QSS-OECT by chemical conditioning and compared to those of the pulse-activated system in Figure 2D. Comparable shape and performance were achieved for both systems, confirming that a good preconditioning toward activation is needed for the systems to work consistently at their maximum performance.

A comparison between the transfer curves for the devices introduced in Figure 2 alongside further *I*_*D*_–*V*_*D*_ output characteristics upon several *V*_*G*_ bias can be found in Figure S5.

Additional experiments were performed to assess the role of the polyelectrolyte bridge. To this end, a control device was built in the same way as the QSS-OECT, but avoiding the polyelectrolyte in the back of the gate, i.e., stacking a paper-Pt electrode on top of the channel without adding Nafion. A comparison of the performance of this control with those of other OECTs is shown in Figure 4 and Figure S10, as well as in Table 1. While the best response is obtained with the LS-OECT, the control device also shows a good sensitivity, particularly in the low concentration range. This enhanced performance of both the LS-OECT and the control is likely related to the mobility of the ions—especially the cations—that act in the gating mechanism of the conducting polymer channel. Nevertheless, the control system shows a more erratic performance with time. The use of polyelectrolyte yields lower sensitivity but provides better long-term stability and small footprint. In fact, Figure 4C and D shows the calibration of peroxide using the same sensor on different days after a pulse activation. The shapes of the time trace profiles in Figure 4C, alongside the sensitivity values provided in Figure S11A, prove that the QSS-OECT was reusable and stable up to 6 times for over 2 weeks, delivering consistent sensitivities ranging from 3.2 to 3.6 mA/dec within an overall [−4.5, −3.25] linear range (log. scale). Regarding the reproducibility in the fabrication and performance of these sensors, Figure S11B shows the calibration curve for the detection of H_2_O_2_ calculated from the average of the signal responses of seven different pulse-activated QSS-OECTs. The inset in Figure S11B includes the sensitivity for each one of these devices. Therefore, good and reproducible values for the sensitivity were achieved, with an average of 3.5 ± 0.3 mA/dec for concentrations between 100 and 1000 μM. Under these conditions, a limit of detection (LOD) of 15 μM is obtained (Table 1).

**Figure 4:**
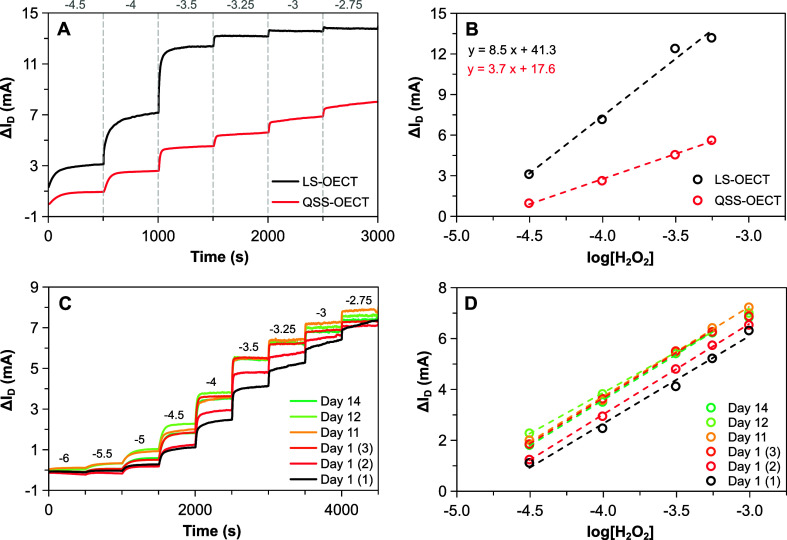
Comparison of the baseline subtracted *I*_*D*_ time traces and calibration curves thereof upon H_2_O_2_ sensing for different OECT configuration (A, B) and regarding sensor stability upon several consecutive tests performed on the same and different days (C, D).

**Table 1:** Analytical Performance of Different H_2_O_2_ OECT-Based Sensors Developed in This Work^a^

System state	Channel	Gate	Sensitivity (mA/dec)	LR	N
LS	PEDOT	Pt/Nafion	8.5	[30, 600] μM	–
Control	PEDOT	Pt	6.6	[30 μM, 1 mM]	–
					
QSS	PEDOT	Pt/Nafion	3.5 ± 0.3	[0.1, 1] mM	7
QSS (drop)	PEDOT	Pt/Nafion	3.2	[0.6, 10] mM	–
QSS (flow)	PEDOT	Pt/Nafion	3.3	[0.1, 1] mM	–

a LR: linear range; N: number of replicas.

To further characterize QSS-OECT, galvanostatic charge/discharge (GCD) tests were performed. In these experiments, the voltage of the working electrode is monitored while a current step is applied, periodically switching polarity. A purely capacitive behavior yields a sawtooth voltage profile. Deviations from this pattern indicate an underlying charging process, such as redox reactions, that will lead to a pseudocapacitive response.^46–48^ In an extreme case, if the voltage reaches a plateau, the system is controlled by redox reactions, indicating a battery-like regime. Figure 5 shows that the time traces for an LS-OECT and a pulse-activated QSS-OECT in PBS result in a similar pseudocapacitive response, which is due to the rearrangement of the ions and the background redox reactions with solution components. Estimated capacitances of these two systems (Figure S12) show no significant differences. With the addition of H_2_O_2_, the profiles flatten, indicating an increase in the capacitance and—at higher concentrations—a battery-like regime due to the predominant role of the redox reactions. Overall, despite of some differences between the LS- and the QSS-OECTs, these results show that they both show similar underlying mechanisms.

**Figure 5:**
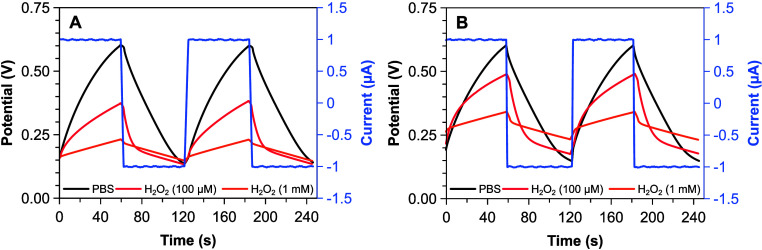
GCD profiles for an LS-OECT (A) and pulse-activated QSS-OECT (B) in absence and presence of H_2_O_2_. Fifth and sixth charge/discharge cycles are shown per each plot.

The advantages of QSS-OECTs are the result of a trade-off between analytical performance and practical features. Some of these characteristics are compared in Figures S14A–C. For example, the pulse-activated QSS-OECT shows higher response times, but it offers enhanced long-term stability. Additionally, the use of Pt electrodes for sensing peroxide may be subject to interferences,^49,50^ such as ascorbic acid. This interference is more severe as the Pt electrode operating voltage increases. In this case, the interference produced by ascorbic acid is shown in Figure S15. While this is a well-known issue that has been reported in the past, it has been also shown that by proper selection of a polyelectrolyte layer this interference can be minimized.^51^

### Sensing in Droplets and Flow Regime

One of the main practical advantages of the QSS-OECT design is the possibility of performing measurements by contact with only one electrode. In conventional electrochemical systems—and in LS-OECTs—the electrical circuit between the electrodes is closed by a liquid electrolyte. Quite often, the sample under analysis fulfills this role. This creates two main issues. First, a larger volume of sample is required to cover all of the electrodes involved in the measurement. Second, the electrical characteristics of the cell—impedance, capacitance, etc.—become dependent on the sample composition. All of these issues are minimized in the QSS-OECT, offering an attractive solution for performing measurements in low volumes of sample and in continuous flow systems.

To prove this point, the detection of peroxide was performed in single drops of 1 μL. The results of these determinations are shown in Figure 6A. The transient signals observed correspond to the decomposition of peroxide on the surface of the gate electrode. The sensitivity under these conditions is 3.2 mA/dec in a range of 0.5 to 10 mM. The system shows a stable response, with a signal that then returns to the baseline after 120 s (Figure S16).

**Figure 6:**
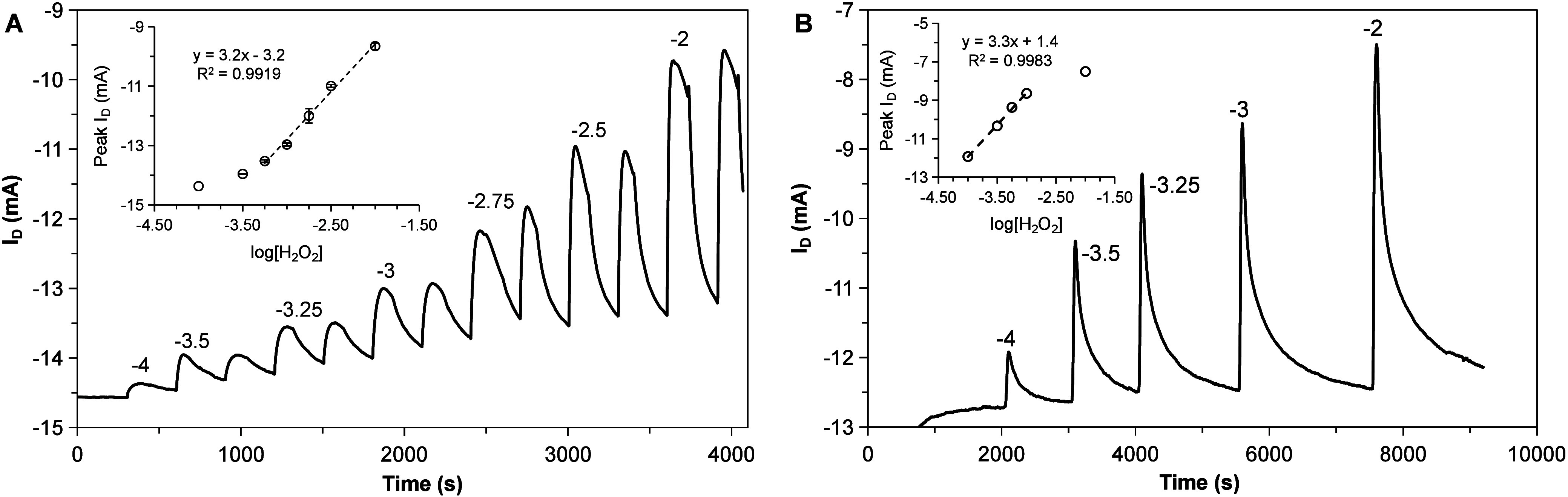
Time traces and calibration curves (insets) for the sensing of H_2_O_2_ in individual droplets (A) and flow conditions (B). Droplet volumes of 1 μL were cast for (A). A flow rate of 50 μL/min was used to irrigate the sensor in (B).

Flow systems also show significant challenges in traditional electrochemical devices since in open cell configurations controlling the flow to cover homogeneously all of the electrodes is challenging and is subject to problems such as bubbles, which disrupt the signal and introduce noise. In the QSS-OECT, the need to cover only one electrode simplifies the hydrodynamic characteristics of the cell, making the arrangement easy to operate. Figure 6B shows the result of the calibration for peroxide under the flow conditions. In this case, a capillary tip discharged directly on top of the transistor gate in an open cell configuration with a flow rate of 50 μL/min. The signals correspond to injection of 1 μL. The sensitivity obtained is 3.3 mA/dec for a log–linear range between 100 and 1000 μM. The calibrations shown in Figure 6 are based on the peak height, although peak areas also show good response (Figure S17). The signals show good reproducibility, with a time to go back to baseline of approximately 160 s (Figures S18–S19). The QSS-OECT proved to be stable upon tests performed on two different days, with sensitivities comparable to those in solution. Thus, QSS-OECTs prove to be promising reusable platforms for a wider range of sensing applications with a reduced sample volume.

## Conclusion

The advantages and drawbacks of using compact paper-based QSS-OECTs as chemical sensors are discussed in this work. Thus, a simple handicraft approach to manufacture thick-film QSS-OECTs with vertical stacking has been introduced by facile printing fabrication. The devices were further characterized then assessed upon the detection of H_2_O_2_ as an analyte of great interest in many (bio)­chemical sensing applications. However, coherent sensing traces were obtained only after reusing the sensor for several runs. Different activation approaches were therefore studied in this work, and a new activation methodology based on a pulsing *V*_*G*_ bias is reported. After a successful activation, the reproducibly fabricated sensors delivered high transconductance values, which accounted for high and consistent sensitivity values when detecting H_2_O_2_. Regarding their operation, the QSS-OECTs proved to be reusable and displayed outstanding performance in terms of sensitivity and linear range. Moreover, the systems effectively worked in single droplet assays and under a flow regime, showing promising perspectives for their integration and usability in future low-cost and distributed sensing platforms, point-of-care applications, and smart wearable devices.

## Supplementary Material



## Notes

Additional experimental and sensor details, materials, and methods, including supplementary experimental assessments (PDF)
