# Supplementary material for: Compact Paper-Based Quasi-Solid-State Organic Electrochemical Transistor (QSS-OECT) for Sensing Hydrogen Peroxide
Source: ACS Appl Electron Mater. 2025 Jul 31;7(15):6791–9. doi: 10.1021/acsaelm.5c00559 (PMC12355906; doi:10.1021/acsaelm.5c00559)
Supplement: Supplementary file 1 [file el5c00559_si_001.pdf]

Supporting Information

Compact Paper-Based Quasi-Solid-State Organic Electrochemical Transistor (QSS-OECT) for Sensing Hydrogen Peroxide

Andrés Alberto Andreo Acosta, Pascal Blondeau, Francisco Javier Andrade*

Department of Analytical Chemistry, Universitat Rovira i Virgili, Carrer Marcel·lí Domingo, 1, 43007, Tarragona, Spain

*E-mail: franciscojavier.andrade@urv.cat.

QSS-OECT Layout and Dimensions

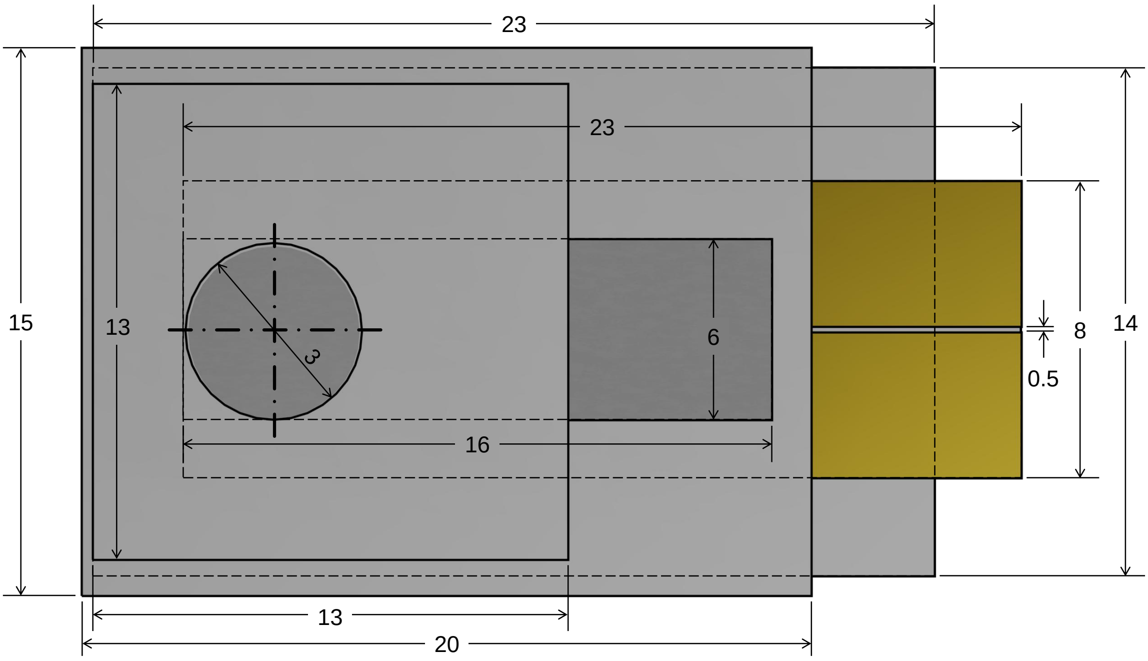

Fig. S1. Top view of a QSS-OECT assembly. Dimensions in millimeters.

Electrical and Electrochemical Characterization

Static resistance was measured between S/D pads using a KEITHLEY 2100 multimeter (Keithley Instruments, Cleveland, OH, USA) in open air. Device was unpowered. Dynamic resistance was determined by measuring the channel current upon steps in V_D from -0.04 to -0.40 V (steps of 0.06 V) in open air. Figure S2 displays the time trace for I_D upon changes in V_D for an LS-OECT (Figure S2A) and a QSS-OECT (Figure S2B). From the inverse of the slope in I_D–V_D output characteristics curve (Figure S2C), we conclude an excellent ohmic behavior with a dynamic channel resistance of 20.8 Ω for the QSS-OECT. Likewise, a value of 23.7 Ω was obtained in the case of the LS-OECT. Reproducibility was further confirmed, with an average static resistance of 12.7 ± 1.4 Ω (Figure S3).

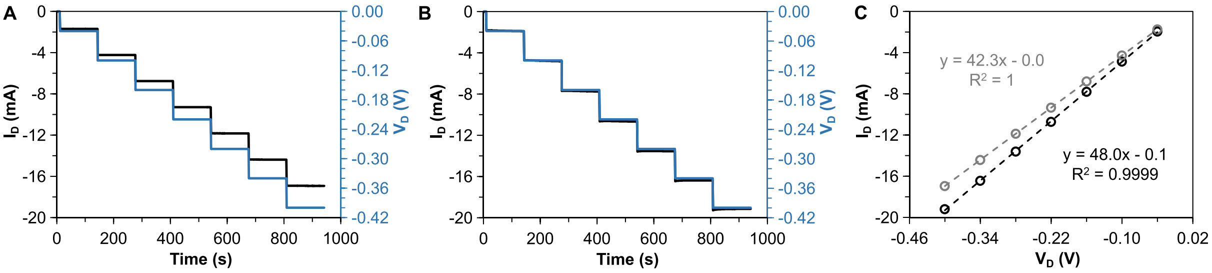

Fig. S2. Time trace for I_D upon changes in V_D for an LS-OECT (A) and a QSS-OECT (B), and I_D–V_D characteristics thereof (C): QSS-OECT (black), LS-OECT (gray).

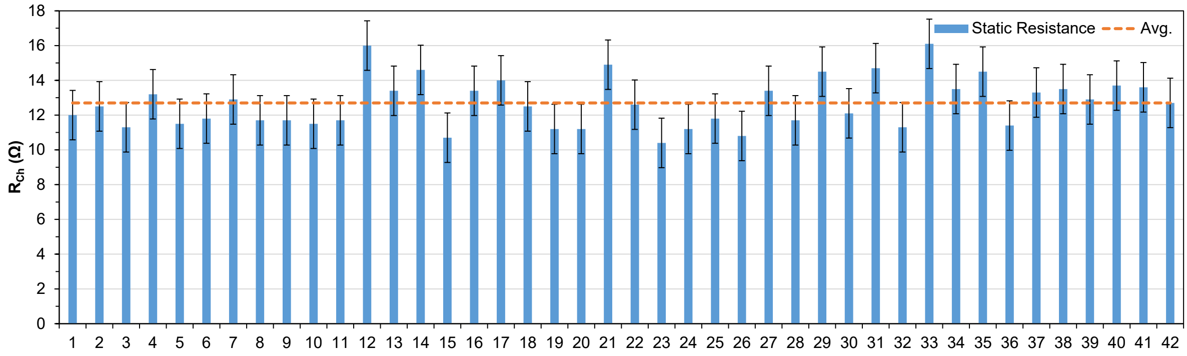

Fig. S3. Reproducibility of the channel fabrication studied as the deviation of the measured static resistance. Horizontal dashed line refers to the average value, error bars to the standard deviation.

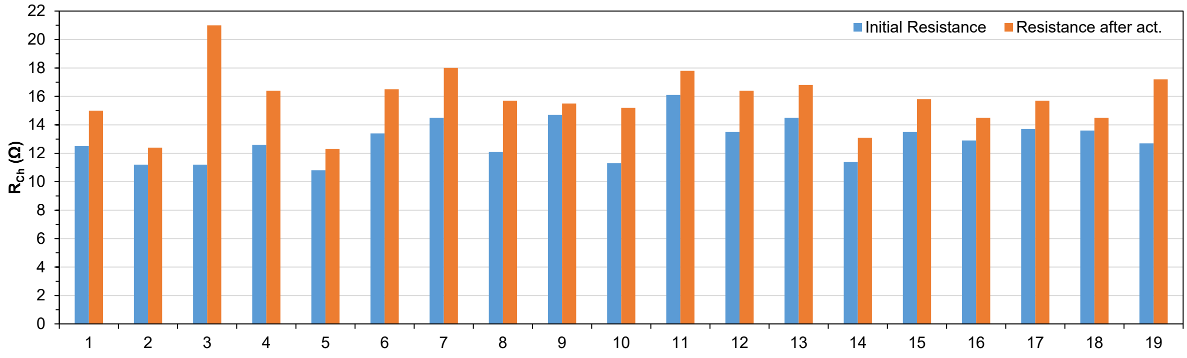

Fig. S4. Values for the measured channel static resistance before (left) and after (right) the pulsing activation. No major changes are encountered, thus the sensor was not damaged by the activation.

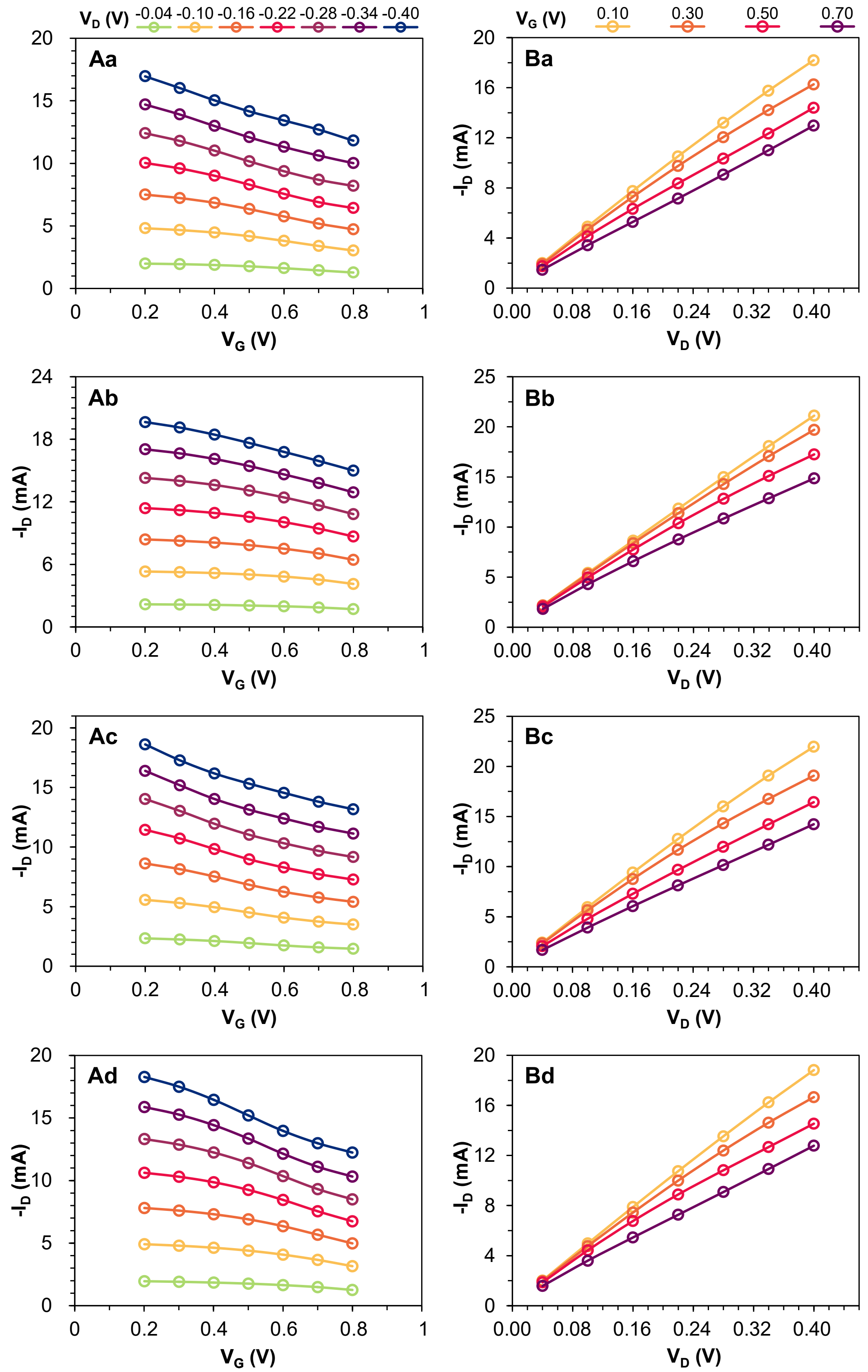

Fig. S5. Comparison of the transfer curves (A) and I_D–V_D output characteristics at several V_G (B) delivered by: (a) an LS-OECT, (b) an as-built QSS-OECT (unconditioned, NC), (c) a chemically conditioned QSS-OECT (first run, CC1), and (d) a pulse-activated (PA) QSS-OECT.

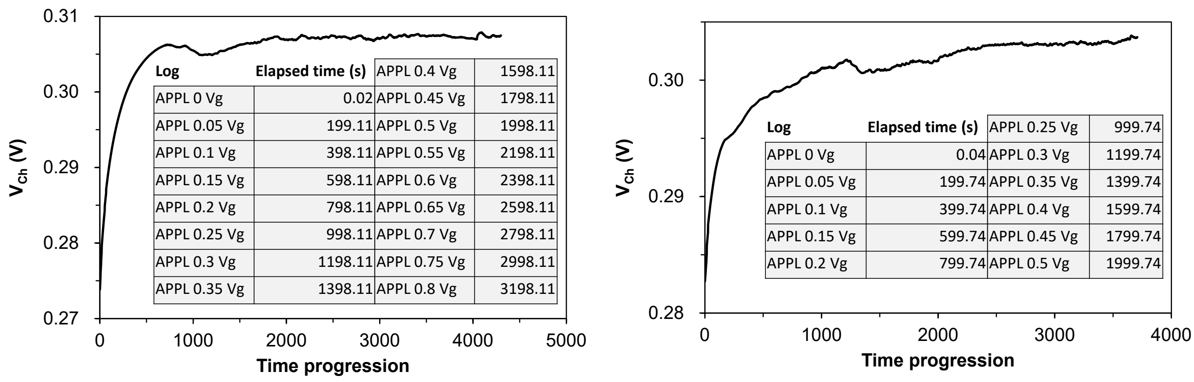

Fig. S6. Time traces for the measured channel voltage upon changes in the gate voltage. No changes were encountered, thus no cross-talk is expected therein.

Preconditioning Assessments and H_2O_2 Sensing

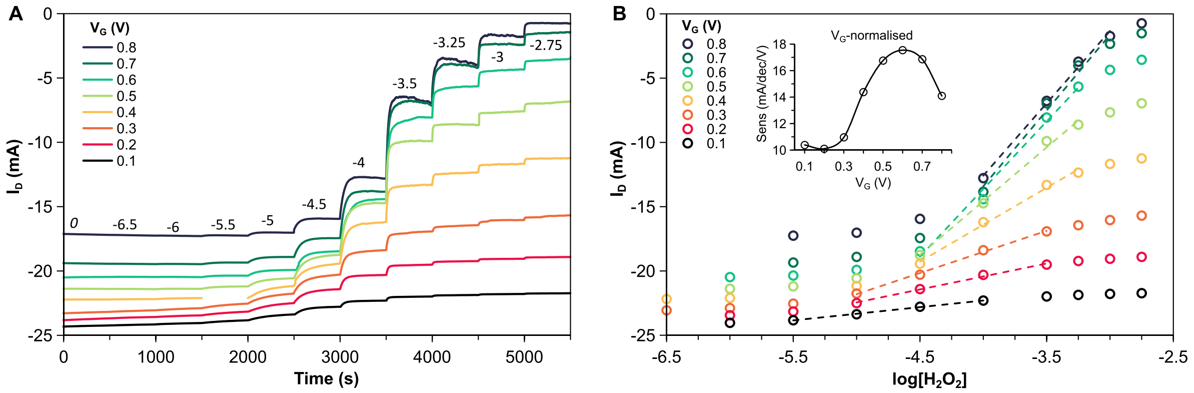

Fig. S7. Time traces (A) and calibration curves thereof (B) for the detection of H_2O_2 at different gate voltages. Inset in the figure refers to sensitivity values normalized by the corresponding applied V_G bias. Linear range shifted toward higher concentrations with higher sensitivity as V_G increases.

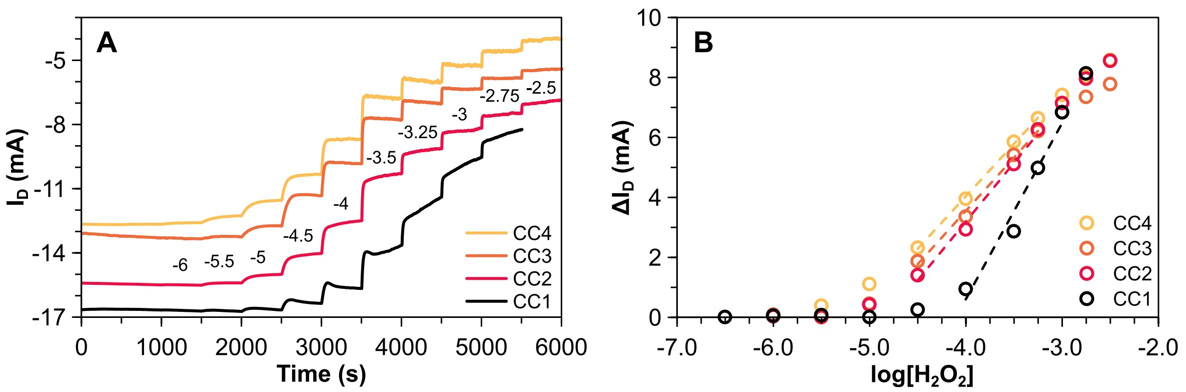

Fig. S8. I_D time traces (A) and calibrations thereof (B) for a chemically conditioned QSS-OECT (CCn, with n test number) upon consecutive H_2O_2 sensing tests at -0.4 V_D, 0.5 V_G.

Table S1. Performance comparison of H_2O_2 OECT sensors in this work with different configuration and activation state.[a] QSS-CC1, QSS-PBS and QSS-NC are excluded due to analytical irrelevance.

System state	Sensitivity (mA/dec)	LR	N	$(t_{response})_{max}$ (s)	$(Drift)_{max}$ (μA/min)	$(\Delta I_i)_{max}$ (mA)
LS	8.47	[30, 600] μM	-	246	97.9	5.2
Control	6.55	[30 μM, 1 mM]	-	185	153.3	4.7
QSS-PA	3.5 ± 0.3	[0.1, 1] mM	7	327	42.0	1.9
QSS-CC4	3.45	[30 μM, 1 mM]	-	138	73.1	1.9
QSS-CC3	3.53	[30 μM, 1 mM]	-	109	82.2	2.1
QSS-CC2	3.93	[30 μM, 1 mM]	-	234	40.0	2.2

[a] LR: linear range; N: number of replicas. LS: LS-OECT; QSS: QSS-OECT; PA: pulse-activated (QSS-OECT); CCn: chemically conditioned by means of H_2O_2, n-th test (QSS-OECT).

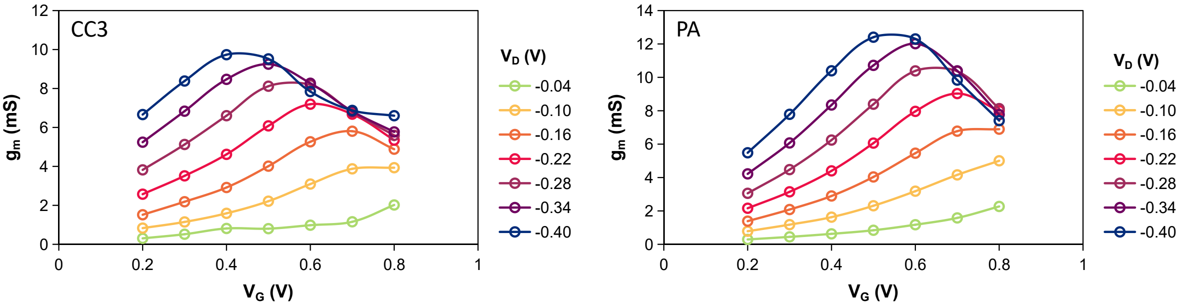

Fig. S9. Comparison of the transconductance profiles for activated QSS-OECTs by means of a chemical activation -third calibration- (CC3, left) and a pulsing activation (PA, right).

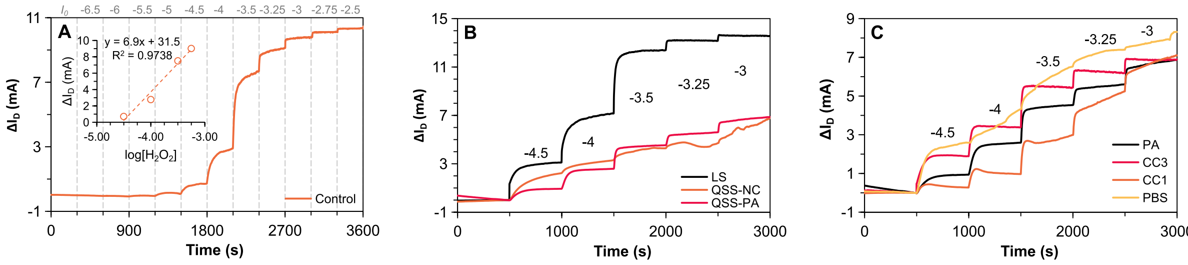

Fig. S10. I_D time traces for for the detection of H_2O_2 with different OECT configurations and different conditioning approaches: Control (QSS-OECT, no Nafion), LS (LS-OECT), QSS (QSS-OECT), PA (QSS, pulse-activated), CC3 (QSS chemically conditioned by means of H_2O_2, third consecutive run), CC1 (QSS chemically conditioned by means of H_2O_2, first test), PBS (QSS, conditioned with PBS), and NC (QSS, non-conditioned). Inset in (A) shows calibration curve for control sensor.

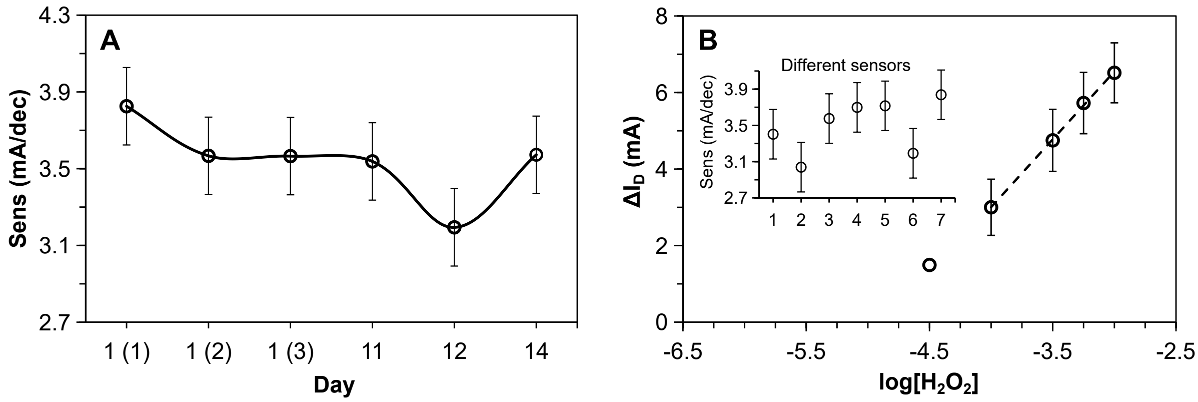

Fig. S11. (A) Sensitivities for consecutive H_2O_2 sensing tests performed with a pulse-activated QSS-OECT on the same and different days. (B) Calibration curve for the detection of H_2O_2 calculated from the average response of 7 different pulse-activated QSS-OECTs. Inset in (B) shows the sensitivities for different replicated sensors.

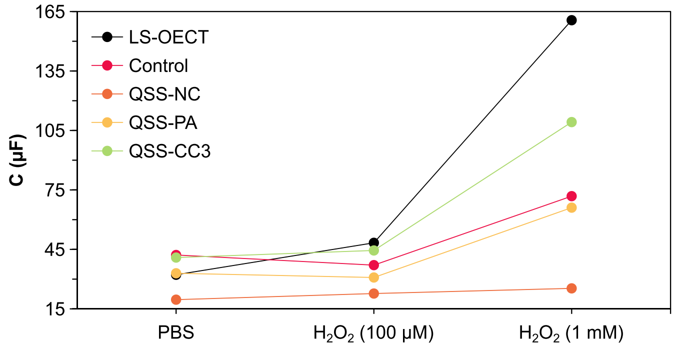

Fig. S12. Capacitance values calculated for the initial linear region of the fifth cycle from the GCD experiments for all the different OECT configurations upon several analyte concentrations. Control sensor without Nafion is included for further comparison.

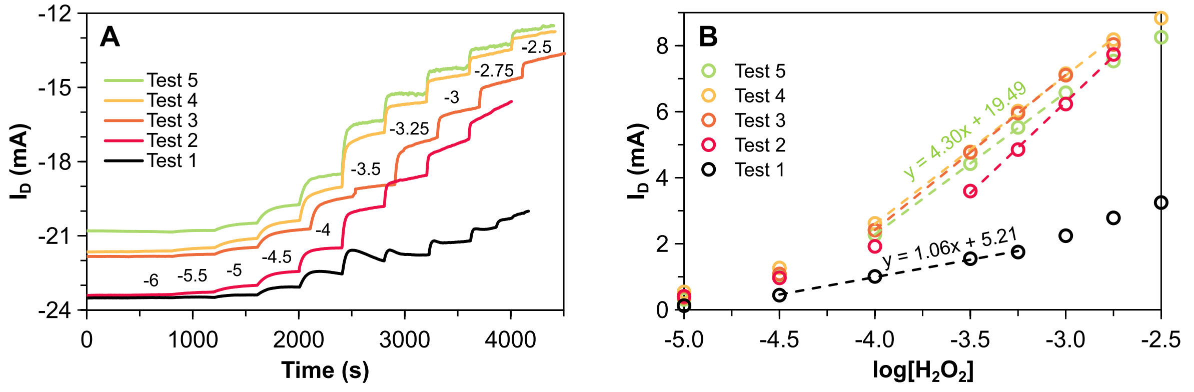

Fig. S13. I_D time traces (A) and calibration curves thereof (B) for an unconditioned QSS-OECT upon consecutive H_2O_2 sensing tests at -0.4 V_D, 0.5 V_G.

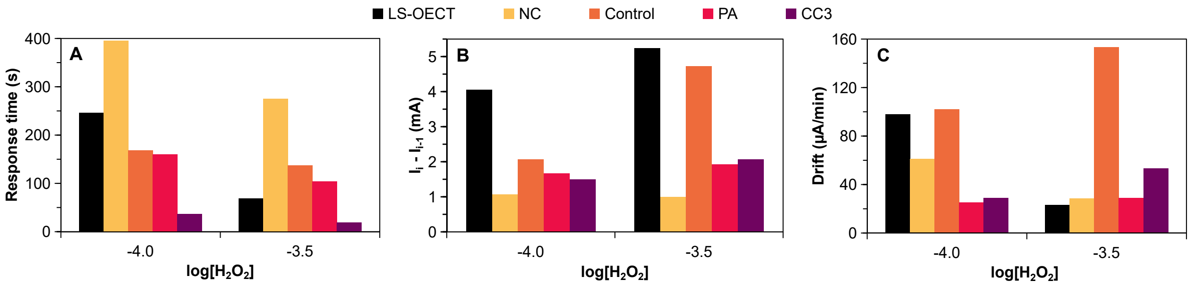

Fig. S14. Performance comparison of H_2O_2 detection for different QSS-OECT conditionings and LS-OECT. Data is expressed as the response time (A), total response (B), and drift (C) following additions of 100 μM (-4, log. scale) and 330 μM (-3.5, log. scale). Response time was calculated as the time to achieve 90% of the steady-state signal upon additions. Control sensor without Nafion is included for further comparison.

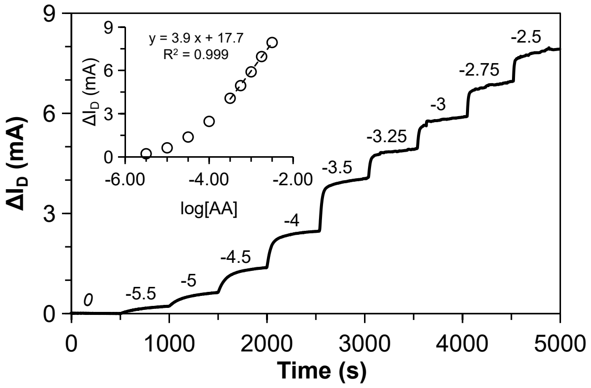

Fig. S15. I_D time trace and calibration curve thereof (inset) for the sensing of ascorbic acid (AA) with a pulse-activated QSS-OECT.

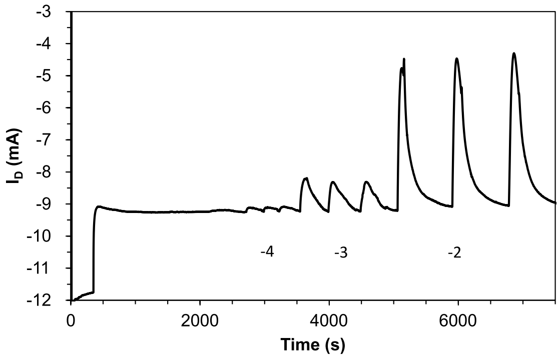

Log[H₂O₂] =	-2	-3	-4
Avg. Peak current (mA)	-4.4 ± 0.1	-8.3 ± 0.1	-9.10 ± 0.01
Baseline recovery (%)	99.8 ± 0.1	99.8 ± 0.2	97.9 ± 0.4
Response time (s)	100 ± 19	105 ± 19	88 ± 22

Fig. S16. Time trace showing further qualitative reproducibility of 1 μL-droplet additions of H_2O_2 at several concentrations.

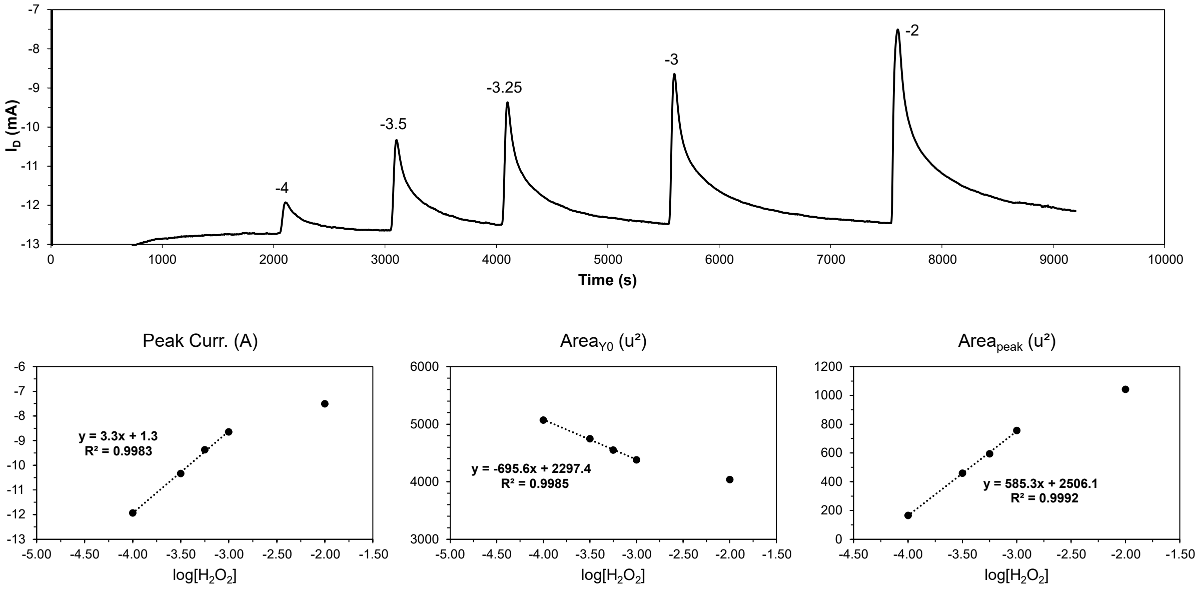

Fig. S17. Time trace and calculated calibration curves thereof for the detection of H_2O_2 in flow conditions. Calibration curves are calculated by considering the channel peak currents (left), and additionally by the peak areas with respect to the $Y = 0$ origin of ordinates (mid) and the peak baseline (right). A minimum baseline recovery of 95% was achieved.

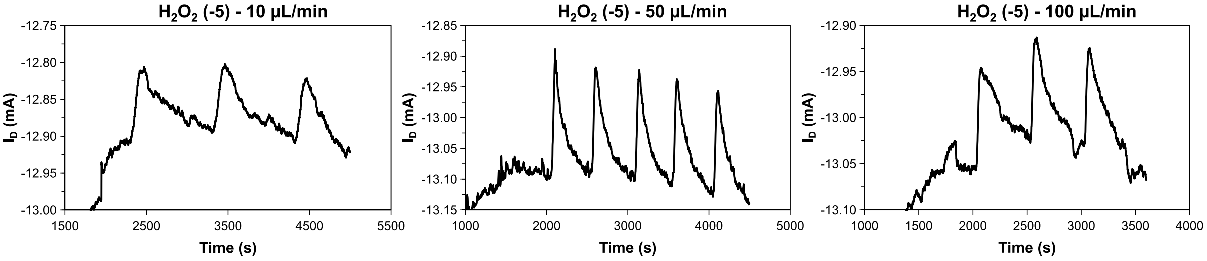

Log[H_2O_2] = -5	10 µL/min	50 µL/min	100 µL/min
Avg. Peak current (mA)	-12.81 ± 0.01	-12.93 ± 0.03	-12.93 ± 0.02
Avg. Peak area | *Y = 0* (u²)	7568 ± 15	5383 ± 17	5812 ± 10
Avg. Peak area | add. baseline (u²)	54 ± 8	29 ± 3	33 ± 3
Baseline recovery (%)	99.7 ± 0.1	99.8 ± 0.1	99.8 ± 0.1
Response time (s)	549 ± 7	135 ± 19	91 ± 10

Fig. S18. Time traces and calculated averages and standard deviations depicting the reproducibility for the detection of H_2O_2 -5 at different flow rates. A minimum baseline recovery of 99% was achieved.

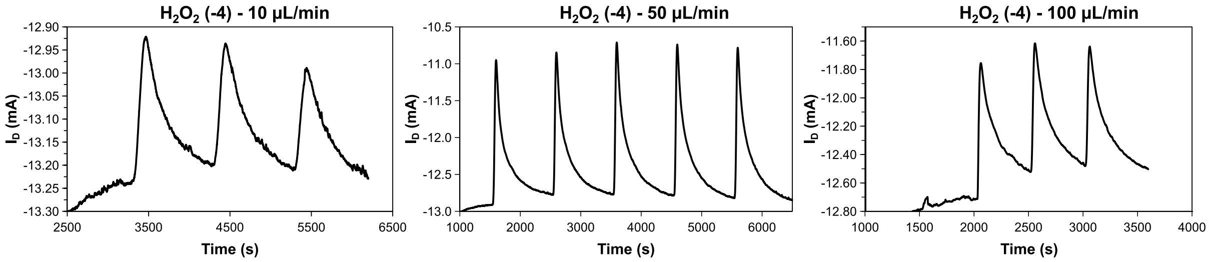

$Log[H_2O_2] = -4$	10 µL/min	50 µL/min	100 µL/min
Avg. Peak current (mA)	-12.95 ± 0.05	-10.8 ± 0.1	-11.67 ± 0.07
Avg. Peak area | *Y = 0* (u^2)	7688 ± 17	4915 ± 28	5452 ± 23
Avg. Peak area | add. baseline (u^2)	110 ± 11	365 ± 12	103 ± 7
Baseline recovery (%)	99.7 ± 0.1	99.1 ± 0.3	98.3 ± 0.2
Response time (s)	530 ± 20	120.9 ± 1.9	72 ± 4

Fig. S19. Time traces and calculated averages and standard deviations depicting the reproducibility for the detection of H_2O_2 -4 at different flow rates. A minimum baseline recovery of 98% was achieved.

Software Framework

All the results were automatically and reproducibly calculated by Excel VBA macros (Microsoft Co., Redmond, WA, USA) available on GitHub.[1] Moreover, KEYSIGHT E3631A programmable PSU (Keysight Technologies, Santa Rosa, CA, USA) was controlled with a Python script to generate the square wave pulses. This code is also available on GitHub.[2]

[1] Andrés Alberto Andreo Acosta. *andriandreo/labExcelForms: CalTest v3.1.* As developed on GitHub. 2022. DOI: 10.5281/ZENODO.5472104.

[2] Andrés Alberto Andreo Acosta. *andriandreo/pyPPS: pyPPS v1.0-beta.* As developed on GitHub. 2023. DOI: 10.5281/ZENODO.8398409.
